# PPero, a Computational Model for Plant PTS1 Type Peroxisomal Protein Prediction

**DOI:** 10.1371/journal.pone.0168912

**Published:** 2017-01-03

**Authors:** Jue Wang, Yejun Wang, Caiji Gao, Liwen Jiang, Dianjing Guo

**Affiliations:** 1 School of Life Sciences and State Key Lab of Agrobiotechnology, The Chinese University of Hong Kong, Hong Kong; 2 Department of Medical Genetics, Shenzhen University Health Science Center, Shenzhen, China; Wuhan Botanical Garden, CHINA

## Abstract

Well-defined motifs often make it easy to investigate protein function and localization. In plants, peroxisomal proteins are guided to peroxisomes mainly by a conserved type 1 (PTS1) or type 2 (PTS2) targeting signal, and the PTS1 motif is commonly used for peroxisome targeting protein prediction. Currently computational prediction of peroxisome targeted PTS1-type proteins are mostly based on the 3 amino acids PTS1 motif and the adjacent sequence which is less than 14 amino acid residue in length. The potential contribution of the adjacent sequences beyond this short region has never been well investigated in plants. In this work, we develop a bi-profile Bayesian SVM method to extract and learn position-based amino acid features for both PTS1 motifs and their extended adjacent sequences in plants. Our proposed model outperformed other implementations with similar applications and achieved the highest accuracy of 93.6% and 92.6% for *Arabidosis* and other plant species respectively. A large scale analysis for Arabidopsis, Rice, Maize, Potato, Wheat, and Soybean proteome was conducted using the proposed model and a batch of candidate PTS1 proteins were predicted. The DNA segments corresponding to the C-terminal sequences of 9 selected candidates were cloned and transformed into *Arabidopsis* for experimental validation, and 5 of them demonstrated peroxisome targeting.

## Introduction

Peroxisome is a small membrane-enclosed organelle widely found in almost all eukaryotic cells [1]. It contains enzymes involved in a variety of metabolic reactions and is known as the site for the production of hydrogen peroxide, hormonal signal molecules, biotin and antibiotics. Enzymes involved in the degradation of peroxides, especially hydrogen peroxide, fatty acids, amino acids, polyamide *etc*., are also found in peroxisomes [2]. In plants, peroxisomes also play important roles in glycolate recycling and amino acid biosynthesis during photosynthesis [3]. Proteins are targeted to the peroxisomes mainly by a conserved peroxisome targeting signal, either type 1 (PTS1) or type 2 (PTS2), which is recognized by the conserved PTS receptors PEX5 and PEX7 respectively. The PTS1 motif is more commonly found in plants compared to PTS2, and it is also called the ‘SKL-motif’ because the tripeptide “Serine-Lysine-Leucine” is the first identified signal [4].

The PTS1 motif, usually consists of an uncharged residue, a basic residue and a non-polar residue at the C-terminal [4], is still widely used for peroxisome targeting protein prediction, localization, as well as genomic and proteomic study [5]. Since most PTS1 motifs can be universally recognized in different organisms [6], the prediction of PTS1 type peroxisomal proteins is usually based on known proteins. However, the short PTS1 motif may not always be sufficient for correct protein targeting. A number of research indicate that the adjacent sequences also play important roles in peroxisomal targeting. Studies in human suggest that the C-terminal 4th~12th amino acids residues are essential in providing backbone and charge [7]. Moreover, the proteins was also targeted to the peroxisome when the C-terminus was extended by 6~7 amino acids with the “SKL” signal at the end [6]. In these cases, the effect of the PTS1 adjacent sequences seem to be important for correct protein targeting.

Some computational tools and web servers for PTS1 protein prediction have been developed in recent years. Georg Neuberger et.al developed a PTS1 signal Predictor [8] in which the silico prediction are solely based on the C-terminal tripeptide PTS1 motif or the short adjacent sequence with less than 14 amino acid residues [7]. Furthermore, for a long time, research on PTS1 proteins mainly focus on animals. For example, PTS1 Predictor, the most cited PTS1 prediction model, only provides animal, fungal and generic version [8]. PredPlantPTS1, the first and only plant-specific PTS1 prediction tool [9], takes the adjacent sequence up to 14 amino acid as input and contains only 60 experimental validated proteins as training data. The possible effect of longer adjacent sequence on correct protein targeting to peroxisome has never been investigated.

In this study, we developed a bi-profile Bayesian (BPB)-SVM based model PPero for plant PTS1 protein prediction. The model extracts and learns position-based amino acid features for both PTS1 motifs and their up to 37 amino acid length adjacent sequence. The performance of PPero was then evaluated by a comparison to other implementations. Finally, a large scale PTS1 protein prediction for Arabidopsis, Rice, Maize, Potato, Wheat, and Soybean proteome was conducted and selected candidate proteins were experimentally validated. The proposed model is anticipated to be a useful resource for computational prediction of PTS1 type proteins in Arabidopsis and other plant species. A web server of PPero is publically available at http://biocomputer.bio.cuhk.edu.hk/PP.

## Materials and Methods

### Data collection and data cleaning

The Arabidopsis protein sequences were collected from TAIR (TAIR10_pep_20101214) [10]. For rice, maize, wheat, soybean and potato, sequences were downloaded from UniProt (UniPrto ID: UP000000763, UP000007305, UP000019116, UP000008827 and UP000011115).

First we searched TAIR and UniProt annotation to look for potential peroxisomal proteins. The annotation in *PeroxisomeDB* and *PeroxiP* were also used as reference [11–12]. The true PTS1 type peroxisomal proteins were manually curated as the positive candidates. Each positive candidate requires the evidence of at least one published paper containing localization study.

Proteins proved to be targeted to nucleus, chloroplast, or mitochondrial etc. by at least two published papers were randomly selected as negative training data. The numbers of positive vs. negative training data ratio was 1:2.

Homology is one of the key factors which may lead to significant bias in the SVM training. We employed multiple alignment, CD-Hit and BLAST search approach to remove the redundant sequences with high similarity (E value < 0.02 in BLAST and no mutual cluster in CD-Hit) [12].

Following the above described steps, 90 positive and 176 negative data were obtained for *Arabidopsis*. For other plant species, collectively 99 positive and 196 negative data were obtained and used for further study.

### Feature extraction and model development

Here let vector *S* = *s*_1_,*s*_2_,…,*s*_*n*_ represent a peptide sequence where *s* denotes amino acid and *1*,*2*,*…*,*n* denotes the position of each amino acid from the C-terminal. *S* belongs to one of the two categories of *C*_*1*_ or *C*_*-1*_, representing the positive data (peroxisomal protein) or negative data (non-peroxisomal protein). For each amino acid *A*_*i*_ in *m* sequences, the prior probability, in other words, the position-specific occurrence is shown as: *P*(A*i*) = *f* (A*i*) /*mi*, where *f* (A*i*) is the function to count the frequency of amino acid A at position *i* in the positive or negative data set respectively. Moreover, Bi-profile Bayes (BPB) method was employed to extract not only positive but also negative data features [13]. According to Bayes’ rule, the posterior probability of *S* is given as:

$$P(c_{1}|S)=\frac{P(S|c_{1})P\left(c_{1}\right)}{P\left(S\right)}$$

$$P(c_{-1}|S)=\frac{P(S|c_{-1})P\left(c_{-1}\right)}{P\left(S\right)}$$

where *P*(*c*_*1*_|*S*) and *P*(*c-*_*1*_|*S*) is given by

$$P\left(S|c_{1}\right)=\overset{n}{\underset{i=1}{\prod }}P\left(s_{i}|c_{1}\right)$$

$$P\left(S|c_{-1}\right)=\overset{n}{\underset{i=1}{\prod }}P(s_{i}|c_{-1})$$

where *s*_*i*_ (*i* = 1,2,…,*n*) should be mutually independent. The *P* (*s*_*i*_|*c*) is the probability of each amino acid at each position in the positive or negative dataset.

Then, we logarithmically reformulated the first two equations. The decision function of *S* is given by the conditional probability of *f*(*S*) = sgn(log(*P*(*c*_*1*_|*S*))-log(*P*(*c*_*-1*_|*S*))). Whether this peptide is peroxisomal targeting or non-targeting is determined by both positive (*c*_*1*_) and negative(*c*_*-1*_) data features.

SVM package *SVM*^*light*^ was employed to build the model [14]. The kernel function and parameters were selected by 5-fold cross validation.

### Performance assessment

In this model, 5-fold cross validation was used to evaluate the performance within each dataset, and the initial subsamples were selected randomly.

Accuracy, Sensitivity, Specificity and Matthews Correlation Coefficient were employed to describe the model performance. The *TP*, *TN*, *FP*, and *FN* represent the true positive, true negative, false positive and false negative, respectively.

$$Acc=\frac{TP+TN}{TP+FP+TN+FN}$$

$$Sensitivity=\frac{TP}{TP+FN}$$

$$Specificity=\frac{TN}{FP+TN}$$

$$MCC=\frac{TP\times TN-FP\times FN}{\sqrt{\left(TP+FP\right)\left(TP+FN\right)\left(TN+FP\right)\left(TN+FN\right)}}$$

### Comparison with other model

Comparison analysis were made between our model and other models using our dataset and others’ original dataset, including those from *PeroxiP*, *PredPlantPTS1* and *PTS1predictor*.

### Genome wide PTS1 protein prediction in plants

Maize, potato, rice, soybean and wheat protein sequence data were collected from UniProt. The last 30 amino acids at the C-terminal were obtained and used as model input. We set the kernel function of SVM to Radial basis function, and the cut-off value was set to 1.5. The training data in all plants were included in the predictions.

For *Arabidopsis*, sequence data were downloaded from TAIR, and only *Arabidopsis* training data PPero_At were selected. The parameters of SVM were the same as previous study.

### Experimental validation

For experimental validation, we randomly chose 12 *Arabidopsis* candidates which are not included in our training datasets from the top listed prediction results. The sequences corresponding to the last 30 amino acids on C-terminal were amplified by RT-PCR with SuperScript III (Thermo Fisher, 18080051) and ligated with a modified plant expression vector pBI221 (https://www.addgene.org/vector-database/1914/) in which the original GUS region was substituted by a yellow fluorescent protein (YFP). Meanwhile, 4-day-old *Arabidopsis* cell suspension culture (cell line *PBS-D)* was treated with 1% (wt/vol) cellulase for protoplast preparation. The recombinant vector was co-transformed together with peroxisome location marker RFP-SKL into *Arabidopsis* protoplast using electroporation method [15]. After overnight culture, the localization of these candidates were observed for the fluorescent YFP and RFP signal under confocal microscope (Olympus FV1000) using the laser wavelength of 488 nm and 568 nm respectively [15]. Finally, the experimentally validated proteins were added into the training data to further improve the model performance.

## Results and Discussion

### Significant amino acid composition bias found in PTS1 type of proteins

For peroxisomal protein prediction in *Arabidopsis* or other plants, we first conducted extensive literature and database search for experimentally validated PTS1 type peroxisomal targeting proteins. In total, 189 candidates from Arabidopsis and 140 from other plants were collected. For negative datasets, we selected 208 and 56 non-peroxisomal proteins from Arabidopsis and other plants respectively. Since a significant number of collected candidates contains at least one orthologous domains, two rounds of data cleaning based on clustering and multiple alignment were employed to eliminate the data redundancy (see methods). After cleaning, two datasets, namely, PPero_At (containing 90 positive proteins and 176 negative proteins in *Arabidopsis*) and PPero_Pt (containing 99 positive proteins and 196 negative proteins in *Arabidopsis*, rice, *Medicago*, *etc*.) were generated. In PPero_Pt, Arabidopsis contributes 67 out of 99 positive candidates and 128 out of 198 negative candidates.

**Fig 1:**
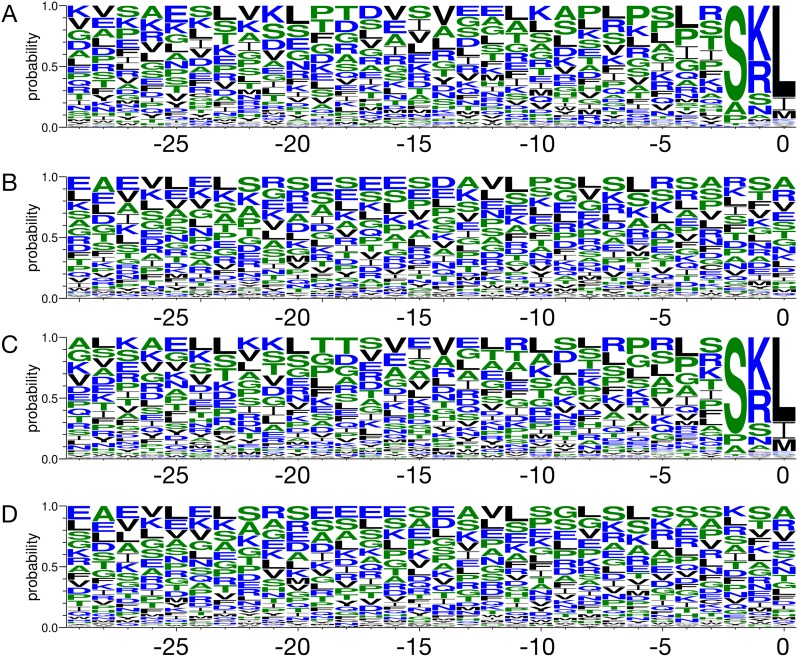
The biased amino acid composition (Aac) in each dataset. (A) peroxisomal proteins in *Arabidopsis*, (B) non-peroxisomal proteins in *Arabidopsis*, (C) peroxisomal proteins in all plants and (D) non-peroxisomal proteins in all plants. The height of each alphabet represents the probability of the corresponding amino acid at each position. The position of the last amino acid from the C-terminus was defined as 0.

We first compared the C-terminal sequences for the non-peroxisomal vs. the peroxisomal targeting protein data in PPero_At and PPero_Pt (Fig 1A, 1B, 1C and 1D). For both datasets, the PTS1 motif (position -2~0) of PTS1-containing proteins showed significant amino acid composition (Aac) preference. As shown in Fig 1A, 1B, 1C and 1D, apparent atypical position-specific Aac bias within position -29~-3 was observed. For example, certain level of Aac bias at position -22~-20, -14, and -6~-4 were found for peroxisomal proteins (Fig 1A) but not for non-peroxisomal proteins (Fig 1B). When data from other plants were added to PPero_At, the Aac bias slightly differs at position -18 and -10 (Fig 1A and 1C). Compared with the data at position -20, -14, and -4 of peroxisomal proteins in plants (Fig 1C), these sites in non-peroxisomal proteins displayed lower Aac preference on average (Fig 1D).

Considering the BPB features of these two datasets, we proposed two versions of model for peroxisomal proteins prediction, namely, PPero 2.0a (Arabidopsis version) and PPero 2.0b (plant generic version). Both models provide brief Web UI and selectable cut-off value.

### Longer adjacent sequence also contributes to model performance

**Fig 2:**
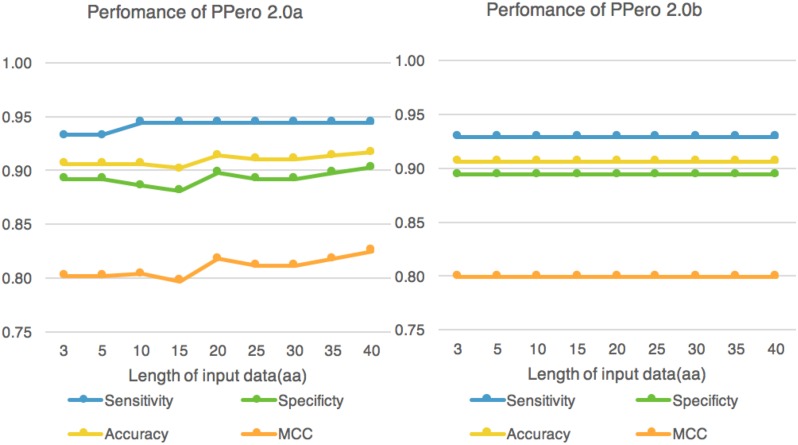
PPero 2.0 performance using different input data length. The length was defined as the number of amino acids from the C-terminus within each protein.

To investigate the contribution of C-terminal adjacent sequences, various lengths of amino acid sequences (3, 5, 10, 15, 20, 25, 30, 35, 40 aa) were used as training data to train the model. As shown in Fig 2, the specificity of PPero 2.0a slightly increased with sequence length whereas the sensitivity remains unchanged. Meanwhile, PPero 2.0b maintained a rather stable prediction power in a range of training data length(Fig 2).

In another preliminary experiment conducted, we found that the -20 to -25 amino acid from the C-terminal may also play key role in peroxisome targeting (S1a–S1g Fig). To include all the possible informative sites, at least 30 amino acids were employed for SVM training and prediction for the following study.

### PPero achieved either high specificity or high sensitivity using selected cut-off value

Given an input sequence, PPero 2.0 will return a numeric value in a range of about -3 to 3. A higher value indicates a higher probability for the sequences to be peroxisomal proteins. A cut-off value is the threshold to determine whether the input protein is peroxisomal targeting or not. As a result, selection of an appropriate cut-off value is one of the key elements that determines the performance of PPero 2.0.

PPero 2.0 achieved either extremely high specificity or high sensitivity depending on selected suitable cut-off values. For PPero 2.0a, the specificity reached 0.987 while the sensitivity decreased to 0.833 under the cut-off value of 1. On the other hand, the sensitivity and specificity were 0.978 and 0.551 respectively under a cut-off value of -1. Similarly for PPero 2.0b, the sensitivity reached 0.990 when the cut-off value was set to -1, and the specificity reached 0.985 when the cut-off value was 1(Table 1). Overall, the MCC of both PPero 2.0a and PPero 2.0b reached the highest levels under a cut-off value of 1. In addition, the default cut-off value of PPero 2.0a and PPero 2.0b were both set to 1.0.

**Table 1:** The model performance comparison under different cut-off values. The MCC of 2.0b with a cut-off -1.5 was not available as the number of true negative decreased to 0.

Cut-off Value	-1.5	-1	-0.5	0	0.5	1	1.5
**Sensitivity(2.0a)**	1	0.978	0.956	0.944	0.944	0.833	0.6
**Specificity(2.0a)**	0.006	0.551	0.875	0.875	0.898	0.987	0.994
**Accuracy(2.0a)**	0.342	0.695	0.902	0.898	0.914	0.936	0.861
**MCC(2.0a)**	0.044	0.518	0.800	0.791	0.818	0.858	0.694
**Sensitivity(2.0b)**	1	0.990	0.949	0.939	0.929	0.808	0.566
**Specificity(2.0b)**	0	0.606	0.869	0.874	0.894	0.985	0.995
**Accuracy(2.0b)**	0.333	0.734	0.896	0.896	0.906	0.926	0.852
**MCC(2.0b)**	N.A.	0.572	0.786	0.783	0.799	0.833	0.671

This model thus provides a flexible tool for research with different purposes through a selectable cut-off value. The higher cut-off value of 1.3 or 1.5 results in high specificity, which is suitable for whole genome prediction. A lower cut-off value, on the other hand, ensures lower false negative rate and is more suitable for functional and localization study of selected proteins. Overall the model achieved good performance, although sometimes the tradeoff may also need to be taken into consideration.

### PPero outperforms other computational models

The performance of PPero 2.0b was further evaluated by a comparison with other peroxisomal protein predicting softwares, including the most cited PTS1 signal Predictor, the plant-specific PTS1 prediction web server *PredPlantPts1*, and the high performance *PeroxiP*. Using the original training datasets and the training methods for each reference model, PPero 2.0b outperformed all the other 3 models based on overall sensitivity and specificity. As summarized in Table 2, the sensitivity and specificity reached 0.929 and 0.894 respectively for PPero_Pt, whereas for other models the values were all less than 0.8. In terms of specificity, the highest of 0.984 and the lowest of 0.640 were obtained for PTS1 Predictor and *PeroxiP* respectively. Moreover, the performance of PPero_Pt and PeroxiP were compared using PeroxiP’s dataset, and similar results was obtained (Table 2).

**Table 2:** The PPero performance comparison with other models. PredPlantPTS1 only provides the result of 10 proteins carrying non-canonical PTS1 motif.

Dataset/Model	Sensitivity	Specificity	Data source or References
**PPero_Pt/PPero 2.0b**	0.929	0.894	http://biocomputer.bio.cuhk.edu.hk/PP
**PeroxiP/PPero 2.0b**	0.651	0.762	http://www.sbc.su.se/~olofe/peroxi/
**PeroxiP/PeroxiP**	0.780	0.640	http://www.sbc.su.se/~olofe/peroxi/ (Emanuelsson, 2003)[12]
**PTS1 Pre/PTS1 Pre**	0.706	0.984	(Neuberger, 2003)[8]
**PredPlantPTS1/PredPlantPTS1**	0.6	N.A.	(Lingner, 2012)[9]

### A large scale peroxisomal protein prediction in plants

The PPero model was adopted for *Arabidopsis* proteome wide prediction. A relative high cut-off value of 1.5 was selected to reduce the false positive rate in this case. Using PPero 2.0a, 735 proteins were predicted as PTS1-type peroxisomal protein candidates. After eliminating the redundant homologous sequences with CD-Hit, 570 proteins remained. In addition, PPero 2.0b was adopted for Maize, potato, rice, soybean, and wheat proteome prediction using a cut-off value of 1.5, and lists of candidate proteins were resulted for each species (S1 File). Apart from sequences with canonical PTS1 motif, a number of proteins with non-canonical PTS1 were also considered as potential peroxisomal proteins, including SLL>, SML> *etc*.

99 predicted peroxisomal proteins in *Arabidopsis* were also cross-validated using Gene Ontology (GO)annotation, which serves as another line of evidence in estimating the accuracy of our model (S1 File). Besides, compared with PlantPrePTS1, 394 proteins could only be specifically predicted by PPero 2.0a. These 394 predicted peroxisomal proteins scored less than 50% when using PlantPrePTS1(S4 File). Among the 395 candidates, some were experimentally validated, e.g. At4G12910 and At3G61960, etc.)[16–17].

### Experimental validation for selected candidates

**Fig 3:**
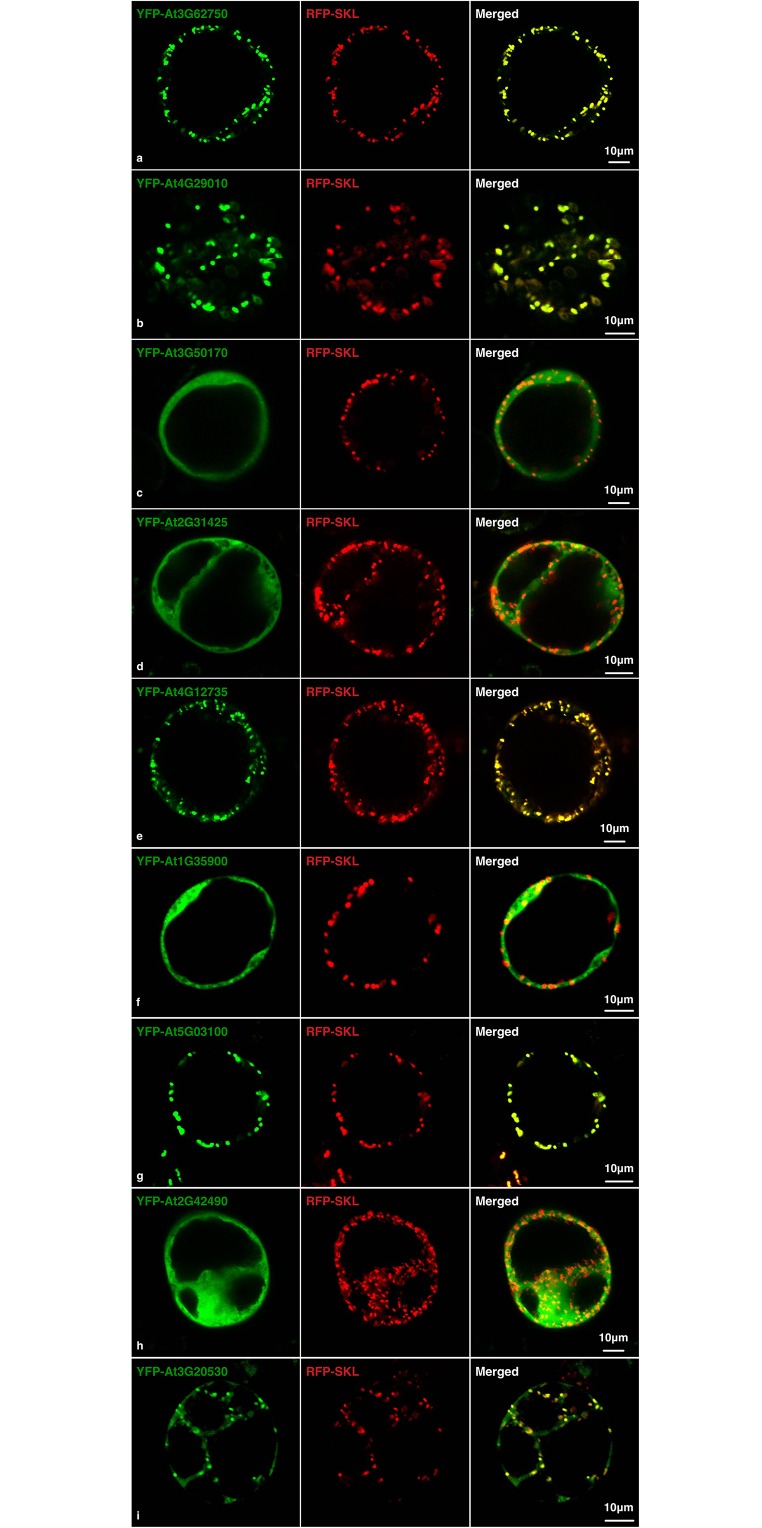
Confocal microscopic observation of YFP labeled prediction candidates (green) and RFP labeled peroxisome location marker (red). AT3G62750, AT4G29010, AT4G12735, AT5G03100 and AT3G20530 showed peroxisomal targeting while AT3G50170, AT2G31425, AT2G42490 and AT1G35900 showed non-targeting.

For experimental validation, 12 candidates from the top prediction list were randomly selected. And 10 of the 12 sequences corresponding to the 30 aa on the C-terminal were successfully cloned and transformed into *Arabidopsis* cell line. After overnight incubation, the candidates fused with YFP showed green fluorescence, whereas the location marker of peroxisome RFP-SKL gave red fluorescence signal. The overlay of YFP signals and the RFP marker indicated the correct peroxisome targeting of the candidates. Of the 10 selected candidates, 9 were expressed and 5 were correctly targeted to the peroxisome (Fig 3). These 5 newly validated proteins were also added into our training datasets.

It is also noteworthy that most predicted candidates with high scores have already been annotated or experimentally studied. For example, 7 out of 10 top ranked Arabidopsis candidates predicted by PPero are annotated as ‘Peroxisome’ related in the TAIR database (https://www.arabidopsis.org), indicating the high efficiency of the proposed model.

## Discussion

Since the first description of PTS1 in 1990s, for a long time, what we understand about the PTS1 signal has been limited to the C-terminal 3 amino acids motif. However, this short motif sometimes may not be enough to guide accurate peroxisome targeting. In recent years, a series of structural information about PTS1-receptor complex were identified in human and *Trypanosoma brucei* by X-ray diffraction and solution NMR [18–20]. The study on PTS1-receptor complex in human implied that some Asn residue of the receptor recognizes and binds to the region beyond the PTS1 motif. For example, Asn 497 can interact with the third amino acid from the C-terminal of the PTS1 containing protein [21], and the 5th amino acid from the C-terminal of the PTS1 proteins shows interaction with Asn 531. Using yeast two-hybrid assay and structure modelling, people found that the -4 to -11 amino acid from the C-terminal were also functional in peroxisome targeting [7]. These studies suggested the critical roles the adjacent sequence may play in receptor and motif recognition. This may also explain why not all of the PTS1 containing proteins are peroxisome targeted. In review of this, it is necessary to take the effect of adjacent sequences into consideration when the prediction model is developed.

The length of effective adjacent sequence in the PTS1 containing proteins is another key factor to be determined. Previous studies in human proved that the -4 to -11 aa were functional in providing charge and backbone for peroxisomal targeting. With that as reference, most of the current models take no more than 12 aa residues as input data. In our preliminary study, a series of adjacent sequences with different lengths were cloned and transformed into *Arabidopsis* cells to examine their effect on correct peroxisome targeting. The PTS1 containing candidates were found to be targeted to peroxisome when the cloned sequence lengths are shorter than 20aa. Interestingly, the targeting abilities were lost when the adjacent sequence lengths are longer than 25aa (S1 Fig). We also found that another protein was targeted to peroxisome only when the sequence length is less than 30aa (Data not shown). The cross-validation results also suggest a slightly better performance when longer input data was used, even though it may lead to more noise. To balance the tradeoffs, we employed a longer sequences of 30aa as the training and input data for the proposed model.

Although the *Arabidopsis* PEX5 shares high sequence homology with PEX5 in human and other species, the amino acid composition of PTS1 motif showed varied specificity. We therefore believe kingdom or even phylum/division specific computational models may facilitate more accurate prediction of peroxisome targeted proteins.

In our experimental validation, 5 out of 9 expressed candidate showed correct targeting. This accuracy is much lower compared to the cross-validation result (>90%). The possible reason for that is we preferably chose hypothetical proteins for experimental validation, e.g. 7 out of the 9 candidates lacks annotations or publications. Furthermore, due to the exclusion of a large number of top-scored annotated candidates, the accuracy of our experimental validation were obviously underestimated. It is reasonable to believe that the real performance of this model could be better reflected if the annotated proteins are also included.

## Supporting Information

S1 Fig The confocal microscopic views of YFP labeled At3g11880 with different adjacent sequence lengths (green) and RFP labeled peroxisome location marker (red). The length is calculated from the C-terminus. A clear peroxisomal targeting was shown when the sequence length is less than 20aa (e~g). Non-targeting was shown when the sequence length exceeds 20aa(a~c). When the length equals to 20, this protein showed dual targeting to peroxisomes and cytoplasm(d).
(TIFF)

S1 File The results from proteome level prediction for rice, maize, wheat, soybean and potato. The predicted peroxisomal proteins and all predicted proteins were listed in MS Excel files.
(ZIP)

S2 File The list of primers used in experimental validation. (CSV)

S3 File The list of training data for PPero 2.0a and PPero 2.0b. All data is listed in fasta and MS Excel format.
(ZIP)

S4 File The list of specifically predicted proteins in *Arabidopsis*. All listed proteins scored 1.5 or above by PPero 2.0a and less than 50% by PlantPrePTS1.
(ZIP)

## Abbreviations

Aac: Amino Acid Composition
BPB: Bi-profile Bayes
GUS: Beta-glucuronidases
PTS1: Peroxisome Targeting Signal type 1
PTS2: Peroxisome Targeting Signal type 2
SKL: Serine Lysine Leucine (the most common used PTS1)
SVM: Support Vector Machine
RFP: Red Fluorescent Protein
RT-PCR: Reverse Transcription Polymerase Chain Reaction
YFP: Yellow Fluorescent Protein
